# *Mosquito*: A Discovery Channel Documentary

**DOI:** 10.3201/eid2308.171064

**Published:** 2017-08

**Authors:** Charles H. Calisher

**Affiliations:** Associate Editor, Emerging Infectious Diseases; Colorado State University, Fort Collins, Colorado, USA

**Keywords:** mosquito, vector-borne infections, Zika, dengue, chikungunya, West Nile virus, viruses, parasites, malaria

The Discovery Channel documentary *Mosquito*, worldwide premiere scheduled for July 6, 2017, is an excellent film intended to educate those who are unaware of the dangers posed by mosquitoes worldwide. Well, not exactly. Mosquitoes themselves pose no great dangers to humans, livestock, or wildlife. It is the viruses or other potential pathogens that mosquitoes carry and can transmit that are the dangers, and they number in the hundreds, if not thousands. Still, *Mosquito* is rather thorough for a 1-hour film.

*Mosquito* provides brief background and informative statements by experts including Anthony Fauci, Director, National Institute of Allergy and Infectious Diseases, National Institutes of Health; Thomas Friedan, former director, Centers for Disease Control and Prevention; Bill Gates, co-chair and trustee, Bill and Melinda Gates Foundation; and Bart Knols, entomologist, author, and entrepreneur. The film delves into recent and past viral and parasitic disease transmission and the roles played by mosquitoes with regard to geographic distributions, natural cycles, epidemiologies, and effects on humans. Although the film describes the terrible illnesses caused by Zika, dengue, chikungunya, and West Nile viruses and malarial parasites, it emphasizes the mosquito vectors that transmit them and the environmental changes that are causing transmission rates to increase.

With proper emphasis on *Aedes aegypti*, *Aedes albopictus*, *Anopheles gambiae*, and other principal mosquito vectors of viruses and parasites, *Mosquito* takes us from the essentials of mosquito breeding to the diseases caused by the agents they carry. The film goes on to show the miserable effects these diseases have on the lives of their victims, along with the economic effects on the victims’ families and communities.

Many vectorborne diseases are geographically isolated and simply ignored by persons outside those areas until such time as they burst forth and affect greater and greater numbers of humans, livestock, or wildlife. A recent example is Zika virus. What the film only implies is that those who control the funds that could be used to understand the potential for disease outbreaks and to predict epidemic spread are not those who are on the battlefield asking for such help. To its credit, however, the film highlights use of bed nets, standard and novel vector-control methods, and the need to develop better universal methods for mosquito control.

The photography is brilliant, albeit somewhat repetitive (hospitalized children and their sad parents), and the dialogue is accurate for the most part. However, to say that *Mosquito* is correct in all aspects would be incorrect; its mention of *Culex* species mosquitoes as proven vectors of Zika virus is, if not incorrect, then premature. Bart Knols, an enthusiastic speaker, has a prominent role in this film, explaining complex processes; he is an expert in malaria control and also a fine teacher of virus transmission by mosquitoes.

